# 1,3-Benzothia­zol-2-amine

**DOI:** 10.1107/S1600536809027561

**Published:** 2009-07-18

**Authors:** Muhammad Altaf, Helen Stoeckli-Evans

**Affiliations:** a Institute of Physics, University of Neuchâtel, rue Emile-Argand 11, CH-2009 Neuchâtel, Switzerland

## Abstract

In the crystal structure of the title compound, C_7_H_6_N_2_S, mol­ecules related by an inversion center are linked *via* N—H⋯N hydrogen bonds involving the amino groups, forming dimers. In turn, these dimers are linked *via* a second N—H⋯N hydrogen bond, forming an infinite two-dimensional network parallel to (011).

## Related literature

For the original powder diffraction study of the title compound, see: Goubitz *et al.* (2001 ▶). For related structures containing the title compound, see: Martínez-Martínez *et al.* (2003 ▶); Padilla-Martínez *et al.* (2003 ▶); Wang *et al.* (2008 ▶). For a description of the Cambridge Structural Database, see: Allen (2002 ▶).
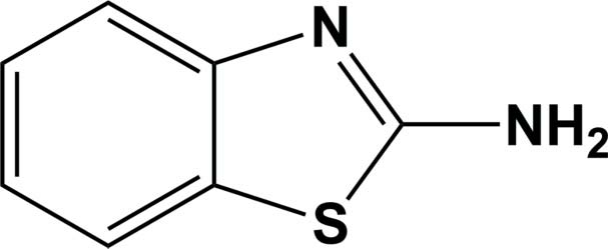


## Experimental

### 

#### Crystal data

C_7_H_6_N_2_S
*M* *_r_* = 150.20
Monoclinic, 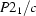

*a* = 14.606 (4) Å
*b* = 3.997 (1) Å
*c* = 11.565 (4) Å
β = 94.47 (2)°
*V* = 673.1 (3) Å^3^
*Z* = 4
Mo *K*α radiation
μ = 0.39 mm^−1^
*T* = 173 K
0.50 × 0.50 × 0.50 mm

#### Data collection

Stoe IPDS-2 diffractometer
Absorption correction: none
2783 measured reflections
1181 independent reflections
1061 reflections with *I* > 2σ(*I*)
*R*_int_ = 0.086

#### Refinement

*R*[*F* ^2^ > 2σ(*F* ^2^)] = 0.052
*wR*(*F* ^2^) = 0.143
*S* = 1.06
1181 reflections
99 parameters
2 restraints
H atoms treated by a mixture of independent and constrained refinement
Δρ_max_ = 0.38 e Å^−3^
Δρ_min_ = −0.31 e Å^−3^

### 

Data collection: *X-AREA* (Stoe & Cie, 2006 ▶); cell refinement: *X-AREA*; data reduction: *X-RED32* (Stoe & Cie, 2006 ▶); program(s) used to solve structure: *SHELXS97* (Sheldrick, 2008 ▶); program(s) used to refine structure: *SHELXL97* (Sheldrick, 2008 ▶); molecular graphics: *PLATON* (Spek, 2009 ▶) and *Mercury* (Macrae *et al.*, 2006 ▶); software used to prepare material for publication: *SHELXL97*.

## Supplementary Material

Crystal structure: contains datablocks I, global. DOI: 10.1107/S1600536809027561/jh2090sup1.cif

Structure factors: contains datablocks I. DOI: 10.1107/S1600536809027561/jh2090Isup2.hkl

Additional supplementary materials:  crystallographic information; 3D view; checkCIF report

## Figures and Tables

**Table 1:** Hydrogen-bond geometry (Å, °)

*D*—H⋯*A*	*D*—H	H⋯*A*	*D*⋯*A*	*D*—H⋯*A*
N2—H2*B*⋯N1^i^	0.83 (2)	2.14 (2)	2.964 (3)	172 (2)
N2—H2*A*⋯N2^ii^	0.80 (2)	2.46 (2)	3.217 (3)	157 (2)

Symmetry codes: (i) 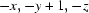; (ii) 
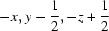
.

**Fig. 1.**
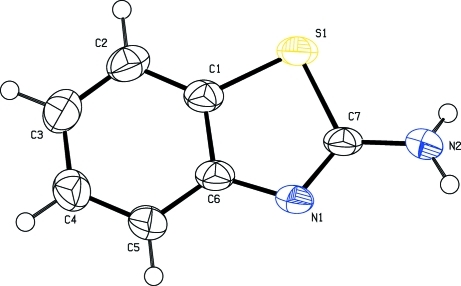
A view of the molecular structure of the title compound, with the displacement ellipsoids drawn at the 50% probability level.

**Fig. 2.**
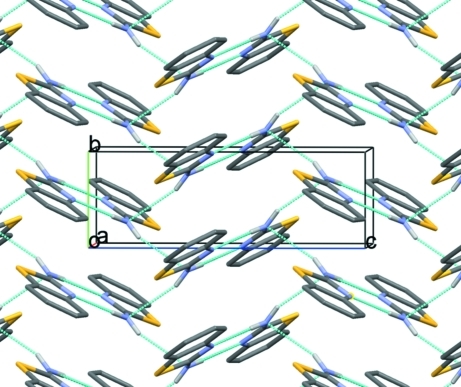
A view along the a axis of the crystal packing of the title compound, illustrating the formation of the centrosymmetric hydrogen bonded dimers and the two-dimensional hydrogen bonded network (see Table 1 for details; the hydrogen bonds are shown a pale-blue dashed lines; H-atoms not involved in hydrogen bonding have been omitted for clarity).

## supplementary crystallographic information

### Comment

The first reference to the crystal structure of the title compound appeared in a study by (Goubitz *et al.*, 2001) on the crystal structure determination of a series of small organic molecules from powder diffraction data. The analysis, using Guinier photographic data, was successful but the final Reitveld refinement gave an *R*_f_ value of only 16.4%. Herein, we report on a single-crystal low temperature analysis (173 K) of the title compound.

A search of the Cambridge Structural Database (Allen, 2002; CSD version 5.30, last update May 2009) revealed the presence of the title compound in a number of co-crystals, for example, with rac-7-oxabicyclo[2.2.1]heptane-2,3-dicarboxylic acid (Wang *et al.*, 2008), and in some (1/1) donor-acceptor complexes, for example, with *N*-benzyl-2-oxo-2*H*-1-benzopyran-3-carboximade (Martínez-Martínez *et al.*, 2003) and ethyl-coumarin-3-carboxylate (Padilla-Martínez *et al.*, 2003).

The molecular structure of the title compound is illustrated in Fig. 1. The geometric parameters are available in the archived CIF and are similar to those in the above mentioned compounds.

In the crystal of the title compound molecules related by an inversion center are linked by N2—H2B··· N1^i^ [symmetry operation: (i) -*x*, -*y* + 1, -*z*] hydrogen bonds to form centrosymmetric dimers (Table 1 and Fig. 2). These dimers are further linked *via* N2—H2A···N1^ii^ [symmetry operation: (ii) -*x*, *y* - 1/2, -*z* + 1/2] interactions to form infinite two-dimensional networks lying parallel to the (011) plane, and stacking along the [100] direction.

### Experimental

Single crystals of the title compound were obtained by recrystallization from methanol of a reagent grade sample.

### Refinement

All the H-atoms could be located in difference electron-density maps. The amino H-atoms were refined isotropically with distance restraints: N—H = 0.82 (2) Å. The aromatic H-atoms were included in calculated postitions and treated as riding atoms: C—H = 0.95 Å with *U*_iso_(H) = 1.2*U*_eq_(parent C-atom).

### Crystal data

**Table:**

C_7_H_6_N_2_S	*F*(000) = 312
*M**_r_* = 150.20	*D*_x_ = 1.482 Mg m^−^^3^
Monoclinic, *P*2_1_/*c*	Mo *K*α radiation, λ = 0.71073 Å
Hall symbol: -P 2ybc	Cell parameters from 42133 reflections
*a* = 14.606 (4) Å	θ = 3.5–25.6°
*b* = 3.997 (1) Å	µ = 0.39 mm^−^^1^
*c* = 11.565 (4) Å	*T* = 173 K
β = 94.47 (2)°	Block, colourless
*V* = 673.1 (3) Å^3^	0.50 × 0.50 × 0.50 mm
*Z* = 4	

### Data collection

**Table:**

Stoe IPDS-2 diffractometer	1061 reflections with *I* > 2σ(*I*)
Radiation source: fine-focus sealed tube	*R*_int_ = 0.086
graphite	θ_max_ = 25.2°, θ_min_ = 3.5°
φ and ω scans	*h* = −17→15
2783 measured reflections	*k* = −4→4
1181 independent reflections	*l* = −13→13

### Refinement

**Table:**

Refinement on *F*^2^	Primary atom site location: structure-invariant direct methods
Least-squares matrix: full	Secondary atom site location: difference Fourier map
*R*[*F*^2^ > 2σ(*F*^2^)] = 0.052	Hydrogen site location: inferred from neighbouring sites
*wR*(*F*^2^) = 0.143	H atoms treated by a mixture of independent and constrained refinement
*S* = 1.06	*w* = 1/[σ^2^(*F*_o_^2^) + (0.1031*P*)^2^ + 0.0494*P*] where *P* = (*F*_o_^2^ + 2*F*_c_^2^)/3
1181 reflections	(Δ/σ)_max_ < 0.001
99 parameters	Δρ_max_ = 0.38 e Å^−^^3^
2 restraints	Δρ_min_ = −0.31 e Å^−^^3^

### Special details

**Table:**

Geometry. Bond distances, angles *etc*. have been calculated using the rounded fractional coordinates. All su's are estimated from the variances of the (full) variance-covariance matrix. The cell e.s.d.'s are taken into account in the estimation of distances, angles and torsion angles
Refinement. Refinement of *F*^2^ against ALL reflections. The weighted *R*-factor *wR* and goodness of fit *S* are based on *F*^2^, conventional *R*-factors *R* are based on *F*, with *F* set to zero for negative *F*^2^. The threshold expression of *F*^2^ > σ(*F*^2^) is used only for calculating *R*-factors(gt) *etc*. and is not relevant to the choice of reflections for refinement. *R*-factors based on *F*^2^ are statistically about twice as large as those based on *F*, and *R*- factors based on ALL data will be even larger.

### Fractional atomic coordinates and isotropic or equivalent isotropic displacement parameters (Å^2^)

**Table:**

	*x*	*y*	*z*	*U*_iso_*/*U*_eq_	
S1	0.17155 (4)	0.19806 (15)	0.24812 (4)	0.0406 (3)	
N1	0.12810 (12)	0.4914 (5)	0.04966 (13)	0.0373 (5)	
N2	0.00290 (14)	0.2707 (6)	0.14135 (17)	0.0427 (7)	
C1	0.25983 (15)	0.3777 (6)	0.17558 (17)	0.0391 (7)	
C2	0.35252 (17)	0.3886 (7)	0.2091 (2)	0.0481 (8)	
C3	0.40994 (17)	0.5406 (7)	0.1364 (2)	0.0523 (8)	
C4	0.37475 (18)	0.6870 (6)	0.0321 (2)	0.0492 (8)	
C5	0.28136 (17)	0.6797 (6)	−0.0011 (2)	0.0435 (7)	
C6	0.22260 (15)	0.5222 (5)	0.07108 (17)	0.0381 (6)	
C7	0.09342 (15)	0.3274 (5)	0.13327 (18)	0.0367 (7)	
H2	0.37610	0.29340	0.28060	0.0580*	
H2A	−0.009 (2)	0.116 (6)	0.182 (2)	0.062 (9)*	
H2B	−0.0377 (18)	0.328 (7)	0.091 (2)	0.063 (9)*	
H3	0.47420	0.54640	0.15710	0.0630*	
H4	0.41550	0.79300	−0.01660	0.0590*	
H5	0.25790	0.78020	−0.07170	0.0520*	

### Atomic displacement parameters (Å^2^)

**Table:**

	*U*^11^	*U*^22^	*U*^33^	*U*^12^	*U*^13^	*U*^23^
S1	0.0458 (5)	0.0516 (5)	0.0240 (4)	0.0062 (2)	0.0010 (3)	0.0025 (2)
N1	0.0421 (10)	0.0481 (10)	0.0218 (8)	0.0010 (8)	0.0038 (7)	−0.0002 (7)
N2	0.0419 (11)	0.0582 (12)	0.0282 (11)	−0.0013 (9)	0.0048 (8)	0.0087 (8)
C1	0.0429 (12)	0.0467 (11)	0.0278 (11)	0.0062 (9)	0.0038 (9)	−0.0062 (8)
C2	0.0472 (13)	0.0581 (14)	0.0376 (12)	0.0064 (11)	−0.0050 (10)	−0.0080 (10)
C3	0.0391 (12)	0.0645 (16)	0.0526 (15)	0.0011 (11)	−0.0001 (11)	−0.0144 (12)
C4	0.0458 (14)	0.0573 (15)	0.0456 (14)	−0.0084 (10)	0.0103 (11)	−0.0114 (10)
C5	0.0454 (13)	0.0546 (14)	0.0310 (11)	−0.0034 (10)	0.0067 (9)	−0.0056 (9)
C6	0.0443 (12)	0.0456 (11)	0.0242 (10)	0.0010 (9)	0.0023 (8)	−0.0057 (8)
C7	0.0425 (12)	0.0459 (13)	0.0215 (10)	0.0039 (9)	0.0017 (9)	−0.0031 (8)

### Geometric parameters (Å, °)

**Table:**

S1—C1	1.747 (2)	C2—C3	1.375 (4)
S1—C7	1.760 (2)	C3—C4	1.402 (3)
N1—C6	1.389 (3)	C4—C5	1.388 (4)
N1—C7	1.303 (3)	C5—C6	1.393 (3)
N2—C7	1.352 (3)	C2—H2	0.9500
N2—H2B	0.83 (2)	C3—H3	0.9500
N2—H2A	0.80 (2)	C4—H4	0.9500
C1—C6	1.410 (3)	C5—H5	0.9500
C1—C2	1.380 (3)		
			
C1—S1—C7	88.64 (10)	N1—C6—C1	115.36 (18)
C6—N1—C7	110.53 (17)	N1—C6—C5	125.68 (19)
H2A—N2—H2B	117 (3)	S1—C7—N1	116.18 (16)
C7—N2—H2B	123.7 (18)	S1—C7—N2	118.60 (16)
C7—N2—H2A	115 (2)	N1—C7—N2	125.1 (2)
C2—C1—C6	122.2 (2)	C1—C2—H2	121.00
S1—C1—C6	109.29 (16)	C3—C2—H2	121.00
S1—C1—C2	128.54 (17)	C2—C3—H3	120.00
C1—C2—C3	118.3 (2)	C4—C3—H3	120.00
C2—C3—C4	120.7 (2)	C3—C4—H4	119.00
C3—C4—C5	121.0 (2)	C5—C4—H4	120.00
C4—C5—C6	118.8 (2)	C4—C5—H5	121.00
C1—C6—C5	119.0 (2)	C6—C5—H5	121.00
			
C7—S1—C1—C2	−179.8 (2)	S1—C1—C6—N1	0.0 (2)
C7—S1—C1—C6	0.41 (17)	S1—C1—C6—C5	−179.83 (17)
C1—S1—C7—N1	−0.79 (18)	C2—C1—C6—N1	−179.8 (2)
C1—S1—C7—N2	−176.53 (19)	C2—C1—C6—C5	0.4 (3)
C7—N1—C6—C1	−0.6 (3)	C1—C2—C3—C4	1.4 (4)
C7—N1—C6—C5	179.2 (2)	C2—C3—C4—C5	−0.6 (4)
C6—N1—C7—S1	0.9 (2)	C3—C4—C5—C6	−0.3 (4)
C6—N1—C7—N2	176.3 (2)	C4—C5—C6—N1	−179.4 (2)
S1—C1—C2—C3	179.0 (2)	C4—C5—C6—C1	0.4 (3)
C6—C1—C2—C3	−1.2 (4)		

### Hydrogen-bond geometry (Å, °)

**Table:**

*D*—H···*A*	*D*—H	H···*A*	*D*···*A*	*D*—H···*A*
N2—H2B···N1^i^	0.83 (2)	2.14 (2)	2.964 (3)	172 (2)
N2—H2A···N2^ii^	0.80 (2)	2.46 (2)	3.217 (3)	157 (2)

Symmetry codes: (i) −*x*, −*y*+1, −*z*; (ii) −*x*, *y*−1/2, −*z*+1/2.
